# Future of Management of Multiple Sclerosis in the Middle East: A Consensus View from Specialists in Ten Countries

**DOI:** 10.1155/2013/952321

**Published:** 2013-12-17

**Authors:** Mohammed Aljumah, Raed Alroughani, I. Alsharoqi, Saeed A. Bohlega, Maurice Dahdaleh, Dirk Deleu, Khaled Esmat, Ahmad Khalifa, Mohammad A. Sahraian, Miklós Szólics, Abdulrahman AlTahan, Bassem I. Yamout, Peter Rieckmann, Abdulkader Daif

**Affiliations:** ^1^King Abdullah International Medical Research Center, King Saud Ben Abdulaziz University for Health Sciences, National Guard Health Affairs, P.O. Box 22490, Riyadh 11426, Saudi Arabia; ^2^Prince Mohammed Ben Abdulaziz Hospital, Ministry of Health, Riyadh 1176, Saudi Arabia; ^3^Division of Neurology, Amiri Hospital Kuwait, P.O. Box 1661, 73767 Qurtoba, Kuwait; ^4^Division of Neurology, Dasman Diabetes Institute, 15462 Dasman, Kuwait; ^5^Clinical Neurosciences Department, Salmaniya Medical Complex, 20525 Manama, Bahrain; ^6^Department of Neurosciences, King Faisal Specialist Hospital and Research Centre, Riyadh 11211, Saudi Arabia; ^7^Department of Internal Medicine, Neurology Section, Arab Medical Center and Khalidi Hospital, P.O. Box 925209, Amman 11190, Jordan; ^8^Department of Neurology (Medicine), Hamad Medical Corporation, P.O. Box 3050, Doha, Qatar; ^9^Merck Serono Intercontinental Region, Dubai 22730, UAE; ^10^Department of Neurology, Damascus Hospital, P.O. Box 10053, Damascus, Syria; ^11^MS Research Center, Neuroscience Institute, Tehran University of Medical Sciences, Tehran 1136746911, Iran; ^12^Division of Neurology, Department of Medicine, Tawam Hospital, P.O. Box 15258, Al Ain, UAE; ^13^Neurology Section, King Khalid University Hospital, College of Medicine, King Saud University, Riyadh 11691, Saudi Arabia; ^14^American University of Beirut Medical Center, Multiple Sclerosis Center, P.O. Box 11-0236, Beirut, Lebanon; ^15^Neurologische Klinik Bamberg, 96049 Bamberg, Germany

## Abstract

The prevalence of multiple sclerosis (MS) is now considered to be medium-to-high in the Middle East and is rising, particularly among women. While the characteristics of the disease and the response of patients to disease-modifying therapies are generally comparable between the Middle East and other areas, significant barriers to achieving optimal care for MS exist in these developing nations. A group of physicians involved in the management of MS in ten Middle Eastern countries met to consider the future of MS care in the region, using a structured process to reach a consensus. Six key priorities were identified: early diagnosis and management of MS, the provision of multidisciplinary MS centres, patient engagement and better communication with stakeholders, regulatory body education and reimbursement, a commitment to research, and more therapy options with better benefit-to-risk ratios. The experts distilled these priorities into a single vision statement: “Optimization of patient-centred multidisciplinary strategies to improve the quality of life of people with MS.” These core principles will contribute to the development of a broader consensus on the future of care for MS in the Middle East.

## 1. Introduction

MS commonly appears in young adults and requires lifelong management, with significant potential for disability among people of working age. Indeed, the World Health Organisation and Multiple Sclerosis International Federation have estimated that about 60% of patients with MS will no longer have full ambulatory function twenty years following diagnosis of the disease [1]. There is a clear need to optimise the care of MS. A group of European experts in MS care recently used a structured process of information sharing and consensus building to define a new vision for optimal MS care in the 21st century [2]. As therapeutic practices and cultural influences vary between regions, it is important that such initiatives be conducted in other parts of the world where MS has a major impact on public health. Accordingly, a group of physicians involved in the care of MS patients from ten Middle Eastern countries recently considered the current and future management of MS within this region.

## 2. Methods

The methodology used previously by a European expert group was adapted for use here [3]. The expert group are all coauthors of this paper and drawn from a panel of experts convened for this purpose (on the basis academic and research history in the field of MS and representation of countries across the Middle East and North Africa) at a closed meeting; additionally, KE acted as Chair and PR (who led the European expert group) acted as Moderator. All suggestions for items of interest were contributed by the Middle-Eastern experts.

Firstly, a list of perceived needs in MS care in the Middle East was generated by participants, in terms of how care for MS might develop in the future, what barriers might prevent the achievement of optimum standards of care, and what factors might drive the change required. Following discussion, this initial list was condensed into a series of principles, which were displayed in view of the group. These items were narrowed down using a voting system in which each participant had five votes which could be allocated in any combination among the principles identified above (e.g., each participant could distribute the votes singly among five different items or, alternatively, up to all five votes could be given to a single item that the individual expert considered to be of major importance). Voting was open and the six principles with the highest total of votes were selected. Participants discussed these further and generated a consensus statement encapsulating their vision for future MS care in the region.

## 3. Overview of the Epidemiology of Multiple Sclerosis in the Middle East

Limited epidemiological data are available from the Middle Eastern countries regarding the prevalence, incidence or natural history (including prognosis and economic impact) of MS, or with regard to the increasing expanding cost of managing the condition. Based on the Kurtzke classification, the Middle East is located in a low-risk zone for MS; however, recent studies suggest a moderate-to-high prevalence in areas within the region (31–55 MS per 100,000 individuals), with an increase in incidence and prevalence in recent years, especially among women [1, 3–8]. Thus, the countries of the Middle East bear a considerable burden of MS. Reliable epidemiological data will be needed for healthcare planning in particular.

## 4. Results of the Consensus Process

The left-hand column of Table 1 shows the twelve core principles identified by experts as important factors underpinning their vision of 21st century care for MS in the Middle East. These encompassed improved communication and contact with diverse stakeholders in care delivery, including patients, healthcare practitioners, and regulators and recognised the importance of personalised care and empowerment of patients. Factors related to treatments for MS were included, with regard to improved therapeutic profiles of interventions, the choice of interventions, the timing of delivery of treatments, and cost. The importance of furthering the research agenda in MS was also recognised, with regard to the need for and design of new clinical studies.

The six core principles arising from the consensus process are shown in the right-hand column of Table 1. It should be noted that questions and discussions during this process led to some merging or modification of original principles that expressed similar or overlapping aspirations for MS care; in all cases, this was achieved by consensus. Accordingly, items were merged relating to communication between stakeholders and patients, to regulatory matters and reimbursement, and to the properties of therapy options. Some other items were made more specific; namely, the experts emphasised the multidisciplinary nature of MS “centres of excellence” and the importance of timely diagnosis of MS in addition to prompt therapeutic intervention where required. The rationale for the six core principles is described separately in the Discussion section.

The six core principles were distilled by consensus into the following vision statement: “Optimization of patient-centred multidisciplinary strategies to improve the quality of life of people with MS.”

## 5. Discussion

### 5.1. Clinical Context for Core Principles

#### 5.1.1. Early Diagnosis and Management of MS

There is no doubt that early diagnosis of MS or its pathological predecessor, CIS, facilitates timely intervention with disease-modifying therapy which, in turn provides better long-term patient outcomes [9, 10]. There is a need to improve the disease recognition and the process and the time of referral for MS patients from primary health care system to specialty care. Moreover, starting therapy earlier is more cost effective than delaying therapy for MS [11]. Training of primary care physicians to recognise the symptoms of MS or CIS and to refer appropriately to a specialist will be required alongside universal access to disease-modifying therapy. Improving access to care was a priority in the 2003 guideline for the management of MS from the UK National Institute for Health and Clinical Excellence (NICE) [12], but even in this developed nation, this goal was described as “aspirational” [13].

#### 5.1.2. Multidisciplinary MS Centres

MS is a complex and multifactorial disease with a major impact on patients, caregivers, and families. The NICE guideline for the management of MS defines the minimum membership of a specialist neurological team as comprising doctors, nurses, physiotherapists, occupational therapists, speech/language therapists, clinical psychologists, and social workers [12]. Additionally, the team may contain expertise in (or have ready access to expertise in) dietetics, liaison psychiatry (to manage comorbid psychiatric conditions), urology (to manage issues related to bladder dysfunction and sexual health), pain management, and ophthalmology. Moreover, effective teamwork is essential to manage the patients' overall condition effectively. The presence of a core team of relevant healthcare professionals within a multidisciplinary clinic will be essential to deliver the multifaceted care necessary to maintain physical functioning and quality of life of the patient with MS.

Publications of detailed audits of the delivery of care in Middle Eastern countries are lacking, in common with other areas of MS care in the regions, as described elsewhere in this paper. The experience of the authors indicated that local healthcare systems are often fragmented and highly variable in regard to resources, quality and accessibility. In general, good care is available with all available approved medication available for prescription; however, the concept of multidisciplinary team-based care is not well established, except in few centres in the region.

#### 5.1.3. Patient Engagement and Better Communication with Stakeholders

Constructive engagement with patients is necessary to support adherence to treatment, which is an important issue in MS and is likely to limit the effectiveness of disease-modifying therapies [14]. Physicians may underestimate the extent of poor adherence to disease-modifying therapies by patients with MS, who frequently take breaks from treatment, and physicians also have limited understanding of the reasons for this behaviour [15]. An earlier expert group of physicians from the Middle East identified education of patients, appropriate management of comorbid conditions that inhibit compliance (such as depression), and the active involvement of specialist nurses and lay support groups as key activities in maintaining optimal adherence to therapy [14]. A recent survey found that emotional support was an important unmet need of patients with MS in USA, which provides an example of an important facet of support that can be delivered by stakeholders other than the physician in the healthcare team [16]. Patients and caregivers have an urgent need for information when diagnosed with MS, and they look to both physicians and nonclinical stakeholders to provide it [17]. The emotional needs of support group personnel also need to be managed, to preserve the quality and availability of support [18]. Improving the self-efficacy of patients with MS has been shown to impact favourably on general aspects of self-care, such as diet [19]. In these ways, positive and constructive engagement between patients and a network of other key stakeholders can improve treatment outcomes in MS.

#### 5.1.4. Regulatory Body Education and Reimbursement

The Middle East is a region of developing nations, with considerable disparities of income within them. Thus, the cost of treatments for MS particularly newer, branded treatments is likely to represent a barrier to care for many patients in the region, as elsewhere, with limited reimbursement for disease-modifying therapies [20]. Moreover, a study in UK showed that higher levels of disability in MS were associated with greater service use and lower quality of life [21].

Provision of adequate reimbursement for treatments for MS will be important to ensure that patients are not faced with insurmountable barriers to adequate care [2]. Regulators and governments will play a key role in determining access to care in MS.  Table 2 provides an overview of the position with regard to reimbursement in two countries from the region, as an example of variations in access to medicines for MS in the region.

#### 5.1.5. Commitment to Research

Treatments for MS are effective in Middle Eastern populations, both in reducing relapse rates patients with clinically established MS [22–26] and in delaying the conversion to MS in patients with a clinically isolated syndrome (CIS) [27, 28]. Nevertheless, a number of important research questions remain. For example, the aetiology of MS and the reasons for its increasing prevalence remain incompletely understood and further knowledge will drive the development of new treatments. Quantitative data on barriers to healthcare for MS in Middle Eastern countries are lacking, but cost/reimbursement, education, and geography are likely to be important: appropriate studies need to be conducted to quantify the importance of barriers to early diagnosis and intervention in the region. A key current aim of treatment in MS is to reduce the rate of progression of disability, and there is considerably more to be achieved in preserving long-term mobility outcomes in this population [5]. In addition, studies to measure and enhance the quality of life of the patient with MS are needed. The clinical characteristics of MS appear to be changing with time, with patients tending to present with milder disease in recent years, which complicates comparisons of efficacy and tolerability of treatments evaluated at different time points [29]. While the evidence base for interferon in MS is extensive, the place of newer disease-modifying agents in MS care is less certain, particularly when patients have progressed on previous treatment (see the section on new therapies, below). In addition, suggestions that interferon *β*_1b_ may have increased longevity in patients with MS need to be evaluated rigorously [30]. Recent research suggests that treatment can be tailored individually, based on characteristics of MS in a given patient, and more research is needed here [31]. Only more randomised, head-to-head trials can fill the gaps in our knowledge regarding the therapeutic profiles of available interventions and of new interventions yet to be introduced.

While a comprehensive review of unmet needs is beyond the scope of this paper, the examples described above provide some areas where new research could impact markedly on the future care of MS. Inevitably, research funds will be limited for MS as a relatively uncommon disease. Projected research funding for the USA in 2013 estimates that *£*121 million will be allocated to MS in that country, with funding for cancer research 45-fold higher, funding for research onto infectious diseases 32-fold higher and funding for heart disease research 10-fold higher, to provide just a few examples [32]. Nevertheless, an increased commitment to research, including in Middle Eastern populations is essential to address important research questions.

#### 5.1.6. More Therapy Options with Better Benefit: Risk Ratios

Disease-modifying therapies reduce the rate of relapse of MS by about 30% per year, and also reduce the rate of development of sustained disability [33–35]. Also, some studies suggest that currently available disease-modifying therapies may delay secondary progression to progressive MS in patients with relapsing-remitting MS [36]. However, the goal of relapse-free lives for patients with the disorder remains elusive [9]. Interferons are generally well tolerated (the main symptoms are injection-site reactions and influenza-like symptoms), although the need for injection is likely to hinder compliance for some patients, and orally administered agents are becoming available [9, 37, 38]. About half of patients in USA who discontinued disease-modifying therapies did so because of side effects, with about one fifth discontinuing through perceived lack of efficacy [39]. There remains room for improvement in therapies for MS, and achieving evidence-based improvements in this area will be linked closely to maintaining a commitment to research, as described above.

### 5.2. Strengths and Limitations of the Consensus Process

The outcome of the consensus process described above reflects the views of physicians caring for patients with MS in Middle Eastern countries. This represents both a strength and a limitation of the process, in that the principles identified reflect the day-to-day experience of these physicians, but necessarily reflect only the views of those physicians present. Also the voting system was open; this encouraged further discussion which in turn strengthened the consensus achieved. The panel were expert physicians in MS care and an important limitation was a lack of perspective from different members of the multidisciplinary healthcare team. The purpose of our paper is to identify initial priorities relevant to the management of MS and the views of all key stakeholders in the delivery of care will be crucial for implementing strategies to make the care of MS in the Middle East for the 21st century.

## 6. Conclusions

We have identified core principles and we believe that they will underpin the optimisation of care for MS in the 21st century. These encompass commitment to research, improved interactions between patients, different healthcare professionals and regulators, and improved processes for delivery of more effective and better tolerated interventions.

## Figures and Tables

**Table 1:** Outcome of the consensus process: principles that underpinned the expert's vision of optimal MS care in the 21st century.

Initial principles identified by discussion	Core principles following the completion of the consensus process
(i) Commitment to research	(i) Commitment to research
(ii) Regulatory body education	(ii) Patient engagement and better communication with stakeholders
(iii) New endpoints in clinical trials	(iii) Regulatory body education and reimbursement
(iv) Healthcare and social care: personalised care	(iv) More therapy options with better benefit: risk ratios
(v) More therapy options	(v) Early diagnosis and management of MS
(vi) MS centres of excellence	(vi) Multidisciplinary MS centres
(vii) Informed, shared decision-making	
(viii) Better communication between stakeholders	
(ix) Cost and reimbursement	
(x) Drugs with better risk: benefit profiles	
(xi) Early treatment	
(xii) Patient engagement and enablement	

The consensus process included questions and discussions leading to some merging or modification of original principles that expressed similar or overlapping aspirations for MS care; accordingly items in the right column are not identical to those in the left column from which they were derived. See text for further details.

**Table 2:** Examples of reimbursement practices in two Middle Eastern countries.

Country	Reimbursement situation
Kingdom of Saudi Arabia	(i) *Government health scheme:* 100% reimbursement (no cost to patients) for *β*-interferons, natalizumab, and fingolimod (ii) *Private health scheme:* 100% reimbursement (no cost to patients) for *β*-interferons (no reimbursement in private hospitals for natalizumab and fingolimod) (iii) Glatiramer is not currently available for prescription in Saudi Arabia

Egypt	(i) Health insurance systems related to the army, police, students, and so forth fully reimburse treatment costs (this represents around 40% of MS patients) (ii) For patients who can afford 60% of the monthly dose costs of *β*-interferon-1aa and *β*- interferon-1b, the government will reimburse the remaining 40% of the cost on submission of a payment receipt proving that they have already purchased and paid for these medications. In practice, this scheme covers about 30% of MS patients (iii) No reimbursement for the remaining 30% of MS patients

^a^Rebif brand (trade mark of Merck Serono). ^b^Betaferon brand (trade mark of Bayer).
